# Design of New Schiff Bases and Their Heavy Metal Ion Complexes for Environmental Applications: A Molecular Dynamics and Density Function Theory Study

**DOI:** 10.3390/ijms25084159

**Published:** 2024-04-09

**Authors:** Maria Assunta Chiacchio, Agata Campisi, Daniela Iannazzo, Salvatore V. Giofrè, Laura Legnani

**Affiliations:** 1 Dipartimento di Scienze del Farmaco e Della Salute, Università di Catania, Viale A. Doria 6, 95125 Catania, Italy; campisag@unict.it; 2 Dipartimento di Ingegneria, Università di Messina, Contrada di Dio, 98166 Messina, Italy; daniela.iannazzo@unime.it; 3 Dipartimento di Scienze Chimiche, Biologiche, Farmaceutiche ed Ambientali, Università di Messina, Viale Annunziata, 98168 Messina, Italy; salvatorevincenzo.giofre@unime.it; 4 Dipartimento di Biotecnologie e Bioscienze, Università di Milano-Bicocca, Piazza della Scienza 2, 20126 Milano, Italy

**Keywords:** DFT calculations, Schiff bases, heavy metals, mercury, lead, water pollution

## Abstract

Schiff bases (SBs) are important ligands in coordination chemistry due to their unique structural properties. Their ability to form complexes with metal ions has been exploited for the environmental detection of emerging water contaminants. In this work, we evaluated the complexation ability of three newly proposed SBs, **1**–**3**, by complete conformational analysis, using a combination of Molecular Dynamics and Density Functional Theory studies, to understand their ability to coordinate toxic heavy metal (HMs) ions. From this study, it emerges that all the ligands present geometries that make them suitable to complex HMs through the *N*-imino moieties or, in the case of **3**, with the support of the oxygen atoms of the ethylene diether chain. In particular, this ligand shows the most promising coordination behavior, particularly with Pb^2+^.

## 1. Introduction

**Figure 1:**
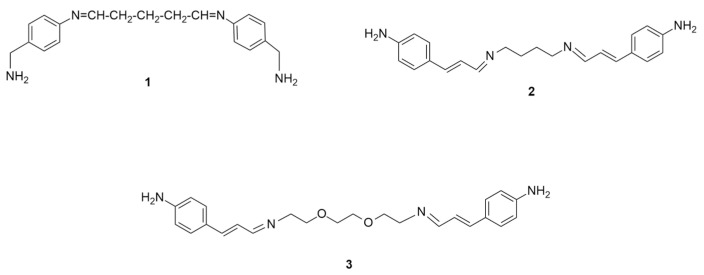
New proposed SBs **1**–**3** as possible ligands for Hg^2+^ and Pb^2+^ ions.

Environmental pollution caused by toxic heavy metals (HMs) represents an ever-growing global problem, due to the negative effects that these contaminants have on the environment, plants, animals and human health [1,2]. Toxic HMs such as arsenic, cadmium, chromium, lead, mercury and zinc, when discharged into water, soils and the atmosphere by agriculture and industry activities or by improper waste disposal, may remain in the environment for a long time, causing an immeasurable amount of biological damage [3,4]. Lead and mercury, in particular, are neurotoxic and cardiotoxic in nature, causing severe health problems to our gastrointestinal, respiratory and central nervous systems [5,6]. The Agency for Toxic Substances and Disease Registry (ATSDR) currently ranks these HMs at the top on their substance priority list, based upon their frequency of toxicity and potential for human exposure [7]. Thus, due to the risks posed by HMs to the environment and the human body, their removal and continuous monitoring in aquatic environment is particularly important. Several technological approaches have been investigated to remove and/or detect these water pollutants, including ion exchange, membrane filtration, chemical precipitation, adsorption and electrochemical approaches [8,9]. However, all the proposed strategies have serious drawbacks, mainly related to their low levels of metal removal, lack of selectivity, high costs and the formation of hazardous residues. Furthermore, a single technology is often not sufficient to solve problems related to the extraction of HMs from contaminated water due to the differences in concentration, pH and physical state of the water-based medium [10]. Therefore, the early and sensitive detection of HMs in wastewater is of the utmost importance to identify the best remediation strategy. Among the different chelating agents used as sensitive elements for the detection of toxic HMs in contaminated waters, Schiff bases (SBs) have been exploited for the environmental detection of metal ions [11,12,13,14,15,16]. These organic compounds, characterized by an imine (-C=N-) functional group formed by the reaction between an amino group and an aldehyde or ketone functionality, have a central role in coordination chemistry and are considered as privileged ligands for complexation with metal ions [17]. The so-formed complexes may exhibit catalytic activity, fluorescence and magnetic behavior, thus providing qualitative and/or quantitative responses [11,18,19]. In this context, systematic theoretical studies can provide useful information about the possible complexation mechanisms and also allow for a comparison with experimental results. Although many studies report the ability of SBs to form stable complexes with HMs, a limited number of Density Function Theory (DFT) calculations have been carried out to investigate the geometries of ligands and their metal complexes [20,21,22]. This paper originates from our previous article focused on the complexation ability of different ligands towards Hg^2+^ and Pb^2+^ ions [23]. In that case, phosphono derivatives were taken into consideration as systems that are able to coordinate toxic metals. The modeling study revealed that the hypothesized N, O chelation mechanism was not correct. In fact, the complexation occurred with two oxygen atoms of the phosphate group of each arm, without involving the nitrogen atoms of the α-aminophosphonic groups. In continuation with these observations, the aim of this work is to design new SBs as possible ligands for HMs using their *N*-imino moieties. Attention was focused on structures **1**–**3** (Figure 1). Ligand **1** is a symmetrical SB with imino moieties distanced by a trimethylene chain and amino groups in benzylic positions, useful for further functionalization. Ligand **2** differs from **1** with the presence of an extra methylene group and the fact that an extended conjugation is possible because the imino groups are not directly linked to the aromatic rings. The same conjugation is also present in ligand **3** that shows an ethylene diether chain, instead of the alkyl one of **1** and **2**.

The conformational preferences of **1**–**3** were detected using Molecular Dynamics (MDs) simulations and Density Functional Theory (DFT) studies. Finally, the possible complexes with Hg^2+^ and Pb^2+^ were optimized with DFT methods, in order to select those with better chelating abilities and address their future development.

## 2. Results and Discussion

### 2.1. Conformational Study of Ligands ***1***–***3***

MD simulations were carried out for 100 ns using explicit water as the solvent. Because of the high degree of conformational freedom of compounds **1**–**3** (Figure 1), to correctly describe their overall behavior and not neglect any possible conformations, the MD study was followed by a cluster analysis. Different conformational families, describing the structures, were located and the most representative ones, populated by more than 10%, were optimized using the widely exploited DFT methods [24,25,26,27].

**Figure 2:**
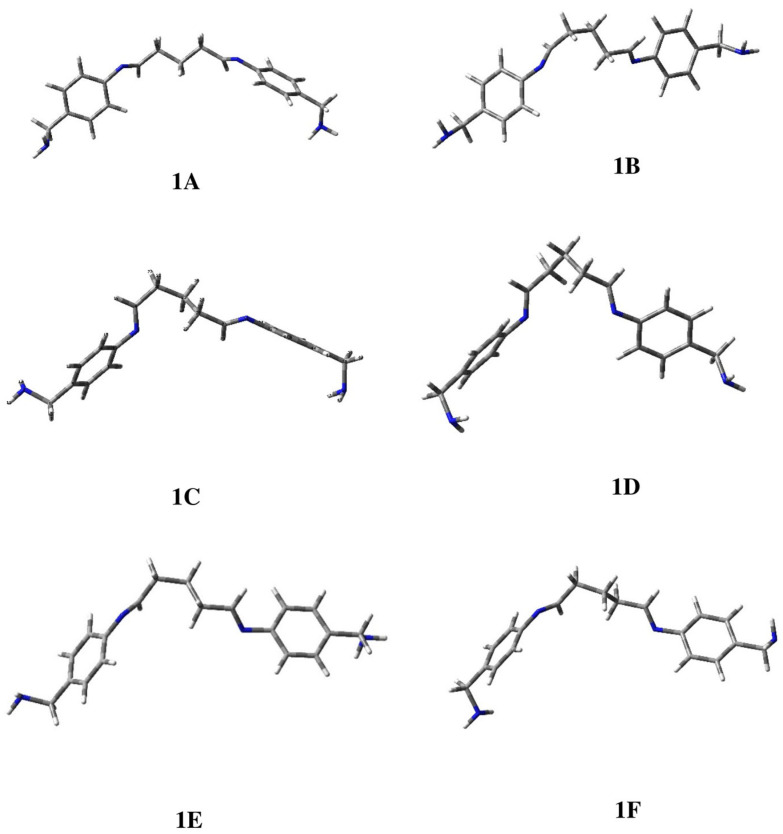
Three-dimensional plots of the located minima of ligand **1**.

For ligand **1** a significant dispersion of the population was observed with six conformational families that resulted in being populated by more than 10%. The selected cluster geometries were optimized at the B3LYP/6-311+G(d) level of calculations and 3D plots of the located minima are shown in Figure 2, while in Table 1, the main geometrical data are summarized, together with the relative energy of each conformation and its percentage contribution to the overall population determined through the Boltzmann equation.

**Figure 3:**
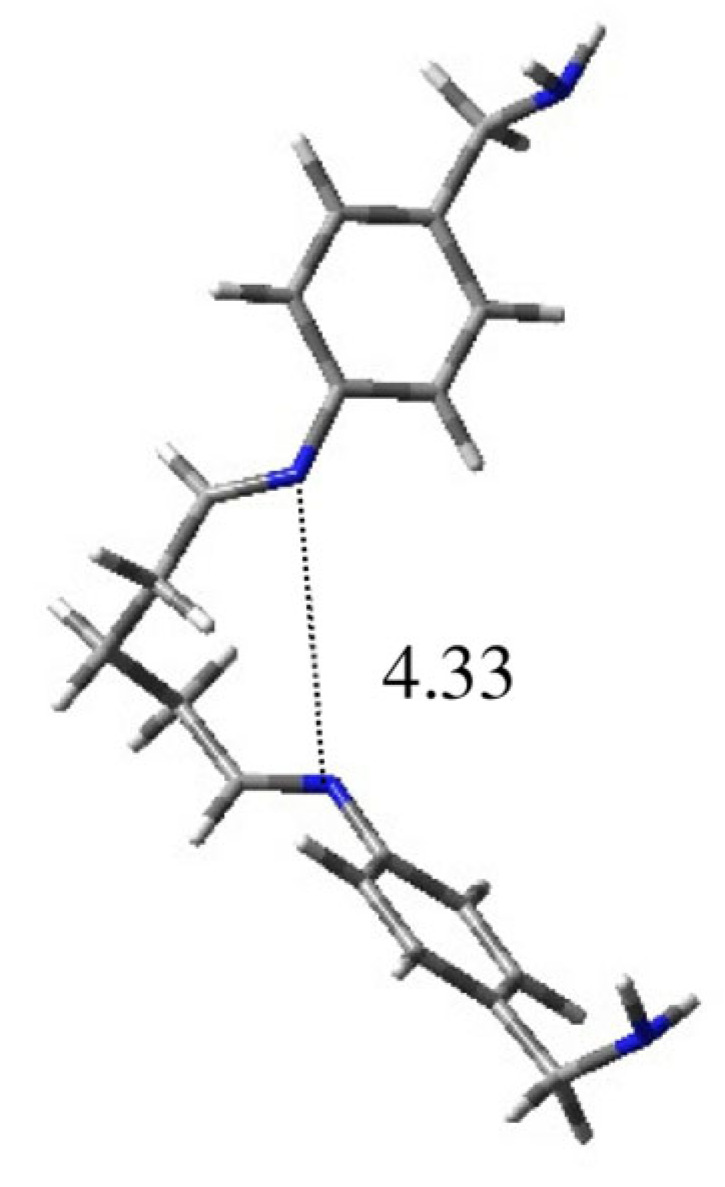
Three-dimensional plot of the lateral view of conformation **1D**. The distance between the nitrogen atoms of the imines is reported in Å.

Conformation **1A** is preferred and accounts for about 27%, while the other populated geometries showed comparable values in the range of 11–15%. As highlighted by the values of the distances (d) between the nitrogen atoms of the imino groups reported in Table 1, only **1D** seems to be productive for the obtainment of a complex. Considering the geometrical features, such as dihedral angles τ3 and τ4, and assuming a gauche orientation, the N atoms will become parallel and at a distance that is theoretically able to allow the chelation of metal ions, as evident by the lateral view reported in Figure 3. Otherwise, in all the other cases this possibility is prevented.

The second proposed ligand is compound **2**, which differs from **1** due to the presence of an extra methylene group and an extended conjugation. This last factor might favor the complexation together making it more easily detectable through different spectroscopic techniques, such as UV spectroscopy.

**Figure 4:**
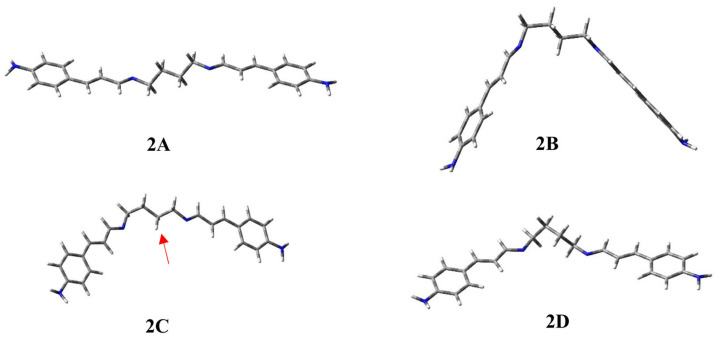
Three-dimensional plots of the located minima of ligand **2**.

The combination of MD and DFT calculations revealed that this ligand prefers an extended geometry like **2A** (Figure 4), which accounts for about 45% of the overall population. This conformation is not productive for complexation because the nitrogen atoms of the imino groups are too far apart (6.24 Å) and point in opposite directions. A second populated conformation (30.5%) is **2B**, which is less stable with respect to **2A** by only 0.68 kcal/mol. In this geometry, the two nitrogen atoms of the Schiff bases are closer (d = 5.44 Å), but, once again, point in opposite directions to each other. Therefore, they are unable to complex the metal ions in this geometry. In conformation **2C**, about 15% of the population, the imino doublets, which have become even closer (d = 5.40 Å) and are pointing in the same direction, may be able to coordinate metal ions. This folded behavior is very similar to that of conformation **1D** of the previous ligand, showing the nitrogen atoms to be parallel. However, the methylene of the spacer, highlighted in Figure 4 by a red arrow, might disfavor complexation by the two imines, replacing one of the nitrogen atoms of the SB in the coordination sphere of the metal.

However, the conformational analysis allows us to locate another minimum (**2D**), which is only about 3% of the population. In this geometry, the spacer carbon chain is arranged so that the two imine nitrogens are 4.8 Å apart and point in the same direction. From a theoretical point of view, conformation **2D** is the only geometry we found that is able to carry out complexation with metal ions using the two imines present in its structure. The main geometrical data are summarized in Table 2, together with the relative energy of each conformation of ligand **2** and the corresponding percentage contribution to the overall population determined through the Boltzmann equation.

**Figure 5:**
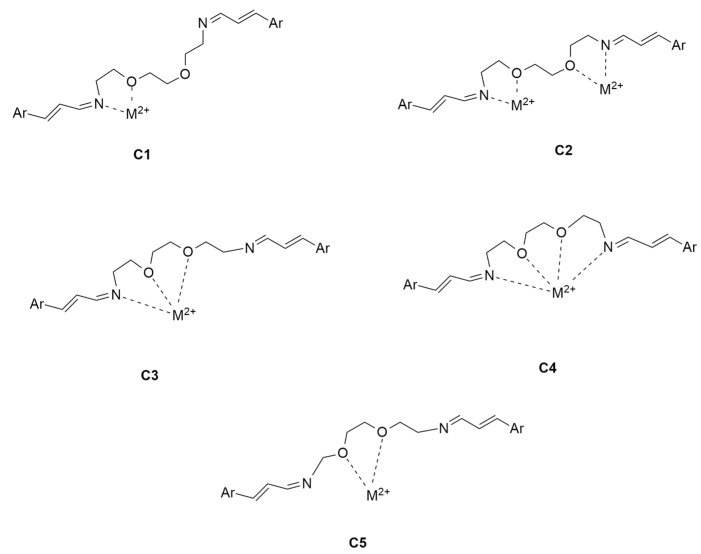
Different possible complexation modes (**C1**–**C5**) of ligand **3** using oxygen and *N*-imino atoms.

Ligand **3** presents with an ethylene diether chain, instead of the alkyl one, between its imine groups and an extended conjugation similar to compound **2**. Theoretically, this structure may lead to different possibilities of complexation, involving both oxygen and *N*-imino atoms, as reported in Figure 5.

**Figure 6:**
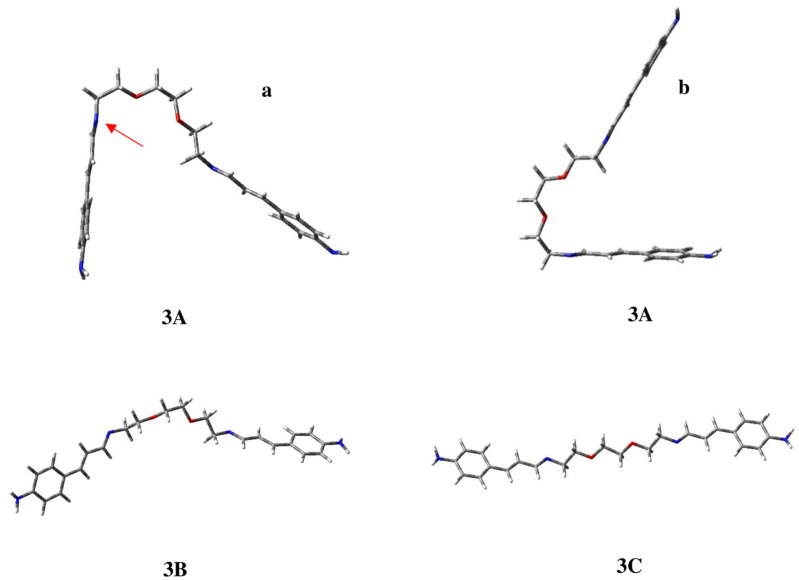
Three-dimensional plots of the populated minima of ligand **3** with conformation **3A** in both frontal (**a**) and lateral (**b**) view.

In order to evaluate the feasibility of the proposed complexation modes, the geometrical preferences of **3** were investigated. The complete conformational analysis shows two preferred geometries that together account for about 92% of the population. The global minimum is **3A** (Figure 6) in which the chain between the Schiff bases is approximately perpendicular to one of the conjugated moieties. The other conjugated portion tends to move away from its twin, in a way similar to a raised level crossing, as evident by the lateral view (**b**) reported in Figure 6. This geometry could be related to a coordination, as in **C3** (Figure 5), involving the two oxygens and the corrected oriented nitrogen atom, highlighted by the red arrow in the frontal view (**a**) in Figure 6.

**Figure 7:**
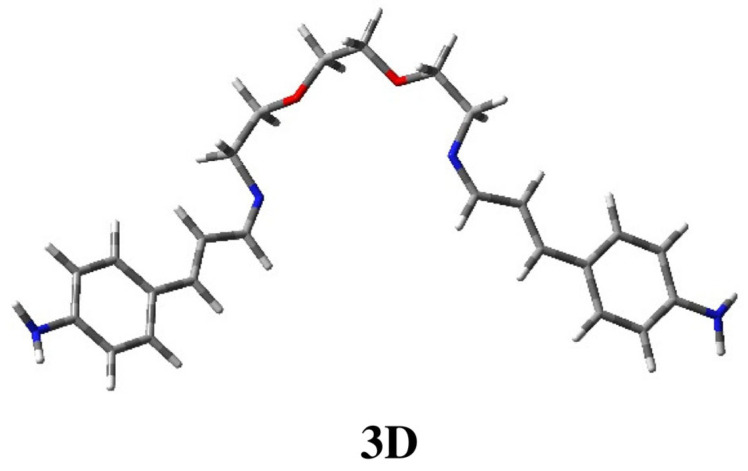
Three-dimensional plot of conformation **3D** of ligand **3**, suitable to provide complexation with the two oxygen atoms and the two imino-nitrogen atoms.

Conformation **3B**, less stable by 0.55 kcal/mol, appears to be similar to **1D**, with the chain spacer in its geometry being analogous to half of a crown ether. In this last case, the complexation of the ion metal can only occur thanks to the oxygen atoms in the chain, as in **C5**. For compound **3** (Figure 5), unlike the other ligands under consideration, the extended arrangement (**3C**), which is not productive for complexation, is less stable by 1.35 kcal/mol and accounts for only 6% of the population. Moreover, it is worth pointing out that calculations also allow us to locate conformation **3D**, reported in Figure 7, which is suitable for tetra-coordination like **C4** (Figure 5). However, this geometry is less stable by 5.74 kcal/mol and appears not to be populated.

The main geometrical data are summarized in Table 3, together with the relative energy of each conformation of ligand **3** and the corresponding percentage contribution to the overall population determined through the Boltzmann equation.

### 2.2. Modeling Study of Complexes of Ligands ***1***–***3*** with Hg^2+^ and Pb^2+^ Metal Ions

**Figure 8:**
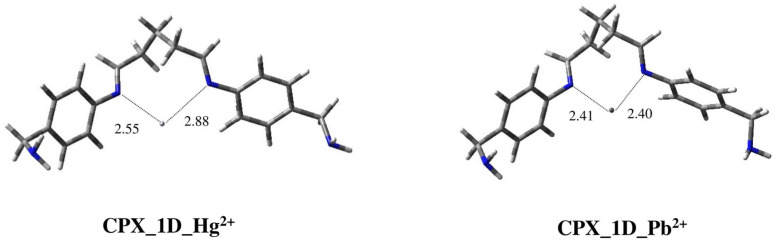
Three-dimensional plots of the located structures of complexes of conformation **1D** of ligand **1** with metal ions Hg^2+^ (**CPX_1D_Hg^2+^**) and Pb^2+^ (**CPX_1D_Pb^2+^**). Distances between *N*-imino atoms and metal ions are reported in Å.

Regarding ligand **1**, as evident from the results shown previously, conformation **1D** is the only one out of the other populated geometries that is suitable for complexation with metal ions, using the *N*-imino atoms. The possible complexes with Hg^2+^ and Pb^2+^ were optimized through DFT calculations at the B3LYP/6-311+G(d) level and the LanL2DZ basis was used for the metals. Figure 8 shows 3D plots of the located structures. 

In the two complexes the metal ions are positioned in the space between the two *N*-imino atoms, parallel to each other. Comparing the two structures, which are very similar, the most evident difference is that, in the case of Pb^2+^, the ion is located closer to the nitrogen atoms in a symmetrical manner, with about the same distance to the imino groups (d_1_(Hg^2+^) = 2.55 Å/d_2_(Hg^2+^) = 2.88 Å vs. d_1_(Pb^2+^) = 2.41 Å/d_2_(Pb^2+^) = 2.40 Å). All these geometrical considerations seem to show a better complexation of Pb^2+^ with respect to Hg^2+^ for this type of ligand.

**Figure 9:**
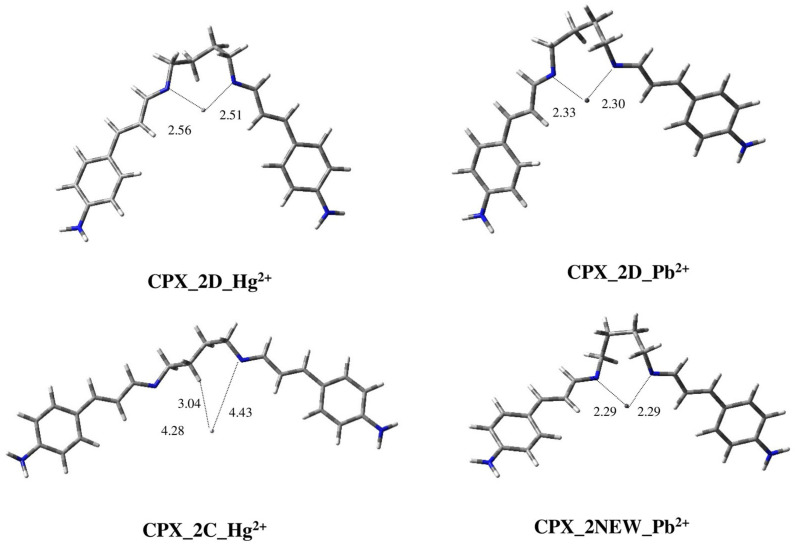
Three-dimensional plots of the located structures of complexes of conformations **2C** and **2D** of ligand **2** with metal ions Hg^2+^ (**CPX_2D_Hg^2+^**, **CPX_2C_Hg^2+^**) and Pb^2+^ (**CPX_1D_Pb^2+^**, **CPX_2NEW_Pb^2+^**). Distances between *N*-imino atoms and metal ions are reported in Å.

In the case of compound **2**, two located geometries, i.e., **2C** and **2D**, may be able to complex the metal ions. Figure 9 shows the 3D-plots of the corresponding complexes with Hg^2+^ and Pb^2+^.

In the case of Hg^2+^, the conformation **2D**, populated by only 3.4%, allowed us to obtain the complex **CPX_2D_Hg^2+^**, which is the most stable one. As evident by the corresponding 3D plot reported in Figure 9, the complexation causes a lowering of the conjugated moieties with respect to the conformation of the free ligand. In fact, in **2D**, the conjugated branches move away in opposite directions with respect to the spacer chain between the Schiff bases. 

Moreover, in **CPX_2D_Hg^2+^**, the metal is placed approximately in the center of the cavity between the two *N*-imino atoms. In this case, in comparison with the complex of the previous ligand (**CPX_1D_Hg^2+^**), the ion is about at the same distance from the two *N*-imino groups (d_1_(Hg^2+^) = 2.56 Å/d_2_(Hg^2+^) = 2.51 Å), probably allowing for a better complexation than **1**.

Instead, geometry **2C** leads to a complex that is less stable, by 5.42 kcal/mol, in which the presence of the methylene group, highlighted by the red arrow in Figure 4, determines the fact that there will be a greater distance between the metal ion and the *N*-imino groups because of the steric hindrance. Furthermore, at the same time, one of the hydrogen molecules in this methylene group experiences an interaction with Hg^2+^ (d_1_(Hg^2+^) = 4.28 Å/d_2_(Hg^2+^) = 4.43 Å/d_H_(Hg^2+^) = 3.04 Å), but this interaction does not translate into the complex being more stable.

In the case of Pb^2+^, as with compound **2**, its complexation with **2D** leads to a geometry in which the ion metal is closer to the *N*-imino atoms than in the complex of the same ligand with Hg^2+^(d_1_(Hg^2+^) = 2.56 Å/d_2_(Hg^2+^) = 2.51 Å vs. d_1_(Pb^2+^) = 2.33 Å/d_2_(Pb^2+^) = 2.30 Å). Instead, the complexation starting from **2C**, after being rearranged through optimization rearranges, produces complex **CPX_2NEW_Pb^2+^**, which has a very similar to geometry **CPX_2D_Pb^2+^**. This could be due to the different size of the ionic radii of the two metals. In fact, the ionic radius of Pb (r(Pb) = 1.19 Å) is greater than that of Hg^2+^ (r(Pb) = 1.02 Å) and so the steric hindrance, determined by the previously highlighted methylene group, does not allow for the complexation of Pb^2+^ with ligand **2** in conformation **2C**.

The new geometry **CPX_2NEW_Pb^2+^**, obtained after optimization starting from the ligand in conformation **2C**, is the global minimum and **CPX_2D_Pb^2+^** results in being less stable by 2.88 kcal/mol with the metal ion, very close to the *N*-imino atoms. In general, ligand **2**, like ligand **1**, seems to have a better complexation with Pb^2+^ rather than Hg^2+^.

In the case of compound **3**, considering the geometries of the free ligand that we located, conformations **3A**, **3B** and **3D** can produce complexation with metal ions on the basis of the complexation modes **C3**, **C5** and **C4** (Figure 5), respectively. 

**Figure 10:**
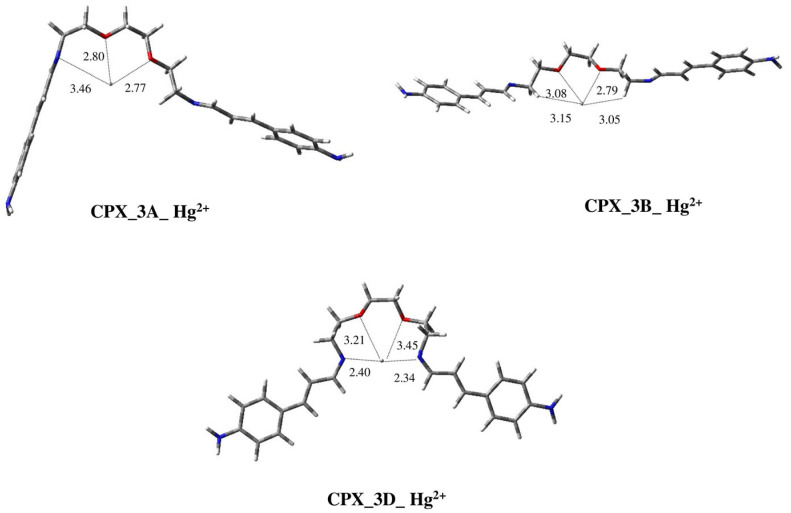
Three-dimensional plots of the located structures of complexes of conformations **3A**, **3B** and **3D** of ligand **3** with Hg^2+^ (**CPX_3A_Hg^2+^**, **CPX_3B_Hg^2+^**, **CPX_3D_Hg^2+^**). Distances between *N*-imino and oxygen atoms and metal ions are reported in Å.

Considering Hg^2+^, the most stable complex we located is **CPX_3D_Hg^2+^** (Figure 10). Compared to the free ligand in conformation **3D**, in this complex, the spacer chain between the imino groups is rearranged, bringing the two N-imino atoms opposite each other at a distance of 4.71 Å. Conversely, in **3D** they are parallel at a distance of 6 Å. In this way, the ion metal fits on the same line as the two nitrogen atoms at about the same distance from each atom (d_1_(N) = 2.40 Å; d_2_(N) = 2.34 Å). Furthermore, the etheric O atoms are also able to efficiently interact with the metal, albeit at a greater distance with respect to the *N*-imino atoms (i.e., d_1_(O) = 3.21 Å; d_2_(O) = 3.45 Å), proving the previously supposed **C4** complexation mode.

The most stable geometry of the free ligand, **3A**, produces a complex in which the metal ion occupies the area delimitated by the spacer chain with, as expected, a **C3** complexation mode (see Figure 5). In this case, the ion metal is closer to the two oxygen atoms with respect to the *N*-imino one involved in the complexation (d_1_(O) = 2.80 Å; d_2_(O) = 2.77 Å; d(N) = 3.46 Å). However, complex **CPX_3A_Hg^2+^** (Figure 10) is less stable than **CPX_3D_Hg^2+^** by 7.90 kcal/mol. This greater energy may be due to the better electronic release effect of the *N*-imino groups with respect to that of the oxygen atoms. 

Finally, a third complex, **CPX_3B_Hg^2+^** (Figure 10), has been located, starting from the conformation **3B** of the free ligand (Figure 6). This geometry, which shows a **C5** complexation mode (Figure 5), presents a coordination with the two oxygen atoms, while the *N*-imino atoms are not involved. In this structure, the metal ion is closer to one oxygen atom compared to the other one ((d_1_(O) = 2.79 Å; d_2_(O) = 3.08 Å). Moreover, one hydrogen atom from each methylene group linked to the imino groups interacts with Hg^2+^ at a distance of about 3 Å, giving further stability to the system. Nevertheless, the energy of this structure is high, being less stable than the global minimum by more than 8 kcal/mol. This result confirms the more favorable effect of the interaction of the metal ion with N-imino groups compared to that from their interactions with oxygen atoms.

Then, the complexes obtained starting with the same geometries of the free ligand, i.e., **3A**, **3B** and **3D**, were located considering Pb^2+^ as the coordinated metal ion.

**Figure 11:**
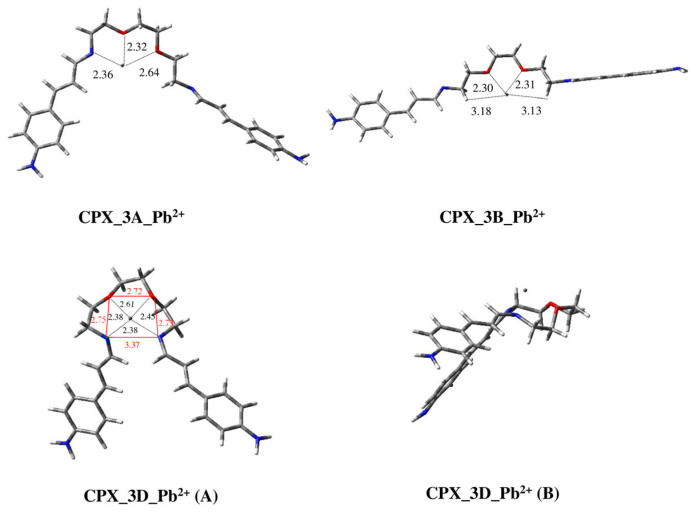
Three-dimensional plots of the located structures of complexes of conformations **3A**, **3B** and **3D** of ligand **3** with metal ions Pb^2+^ (**CPX_3A_Pb^2+^**, **CPX_3B_Pb^2+^** and **CPX_3D_Pb^2+^** in frontal (**A**) and lateral (**B**) view). Distances between *N*-imino and oxygen atoms and metal ions are reported in Å together with distances between heteroatoms.

Conformation **3D** allows a complexation (**CPX_3D_Pb^2+^**) in which the two *N*-imino atoms and the two oxygen atoms are on the same plane, forming the isosceles trapezoid base of a pyramid whose tip is the metal ion (Figure 11). This geometry is very stable because of the **C4** type tetra-coordination (Figure 5) and the shorter distances to the four coordination centers with respect to the same complex with Hg^2+^ (d_1_(O) = 2.61 Å; d_2_(O) = 2.45 Å; d_1_(N) = 2.38 Å; d_1_(N) = 2.38 Å for **CPX_3D_Pb^2+^** vs. d_1_(O) = 3.21 Å; d_2_(O) = 3.45 Å; d_1_(N) = 2.40 Å; d_1_(N) = 2.34 Å for **CPX_3D_Hg^2+^**).

Conformation **3A** leads to a **C3** type Pb^2+^ complex, in which, once again, the interaction with the ion metal seems to be better than that of Hg^2+^ (**CPX_3A_Pb^2+^**, Figure 11). In fact, a comparison between the distance values allows us to confirm the proximity of the metal ion Pb^2+^ (d_1_(N) = 2.36, d_1_(O) = 2.32, d_2_(O) = 2.64) to the coordinated atoms with respect to Hg^2+^ (d_1_(N) = 3.46, d_1_(O) = 2.80, d_2_(O) = 2.77).

Also, conformation **3B** is able to coordinate the metal ion (**C5**, Figure 5), giving a complex very similar to that formed with Hg^2+^ (**CPX_3B_ Pb^2+^**, Figure 11). Once again, the metal ion is closer to the oxygen atoms and, in analogy with **CPX_3B_Hg^2+^**, one hydrogen atom of each methylene that is bonded to the imino groups interacts with Pb^2+^.

Nevertheless, these last two complexes (**CPX_3A_Pb^2+^**, **CPX_3B_Pb^2+^**) are not populated because they are less stable than the global minimum **CPX_3D_Pb^2+^** by more than 20 kcal/mol. 

It is worth pointing out that, although the starting geometry **3D** is not populated, it becomes the only possible geometry that can allow the complex to be formed. Moreover, in the case of **CPX_3D_Pb^2+^**, the ΔE values with respect to the other located conformations (**CPX_3A_Pb^2+^**, **CPX_3B_Pb^2+^**) increase considerably. This may be due to the better **C4** coordination (Figure 5) determined by the shorter distances between the metal ion Pb^2+^ and the coordination centers with respect to what was reported in the case of Hg^2+^.

**Figure 12:**
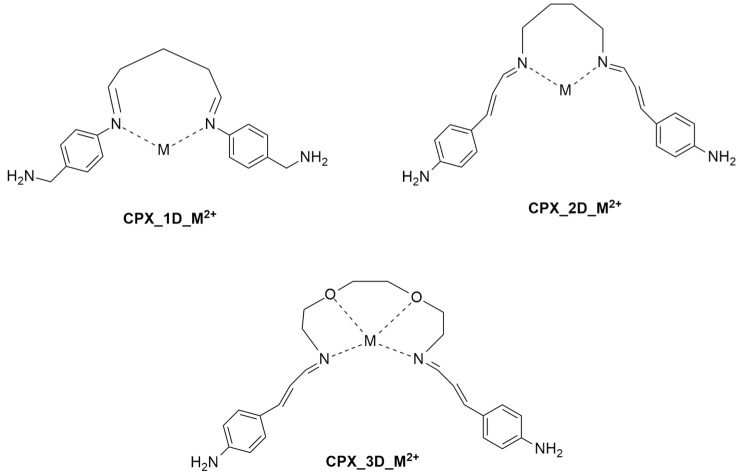
Bidimensional structures of the most promising proposed metal complexes (**CPX**) with ligands in conformation **D** (**1**–**3D**) and metal ions (Hg^2+^, Pb^2+^).

To summarize the results obtained concerning the complexation of **1**–**3** with Hg^2+^ and Pb^2+^, the bidimensional structures of the most promising metal complexes (**CPX**) proposed with ligands in conformation **D** (**1**–**3D**) and metal ions (Hg^2+^, Pb^2+^) are reported in Figure 12, while the energy values of the complexes of the three new SBs we located are reported in Table 4. 

## 3. Materials and Methods

The starting 3D structures of the modeled molecules were built using the program GaussView 6.0.16, a graphical interface of Gaussian16 [28]. Because of their high degree of conformational freedom, the SBs 1–3 have been investigated by MD simulations with the SANDER module of the AMBER20 package [29] using general amber force field (gaff) [30] and RESP atomic charges [31]. The TIP3P model [32] was employed to explicitly represent water molecules. Ligands 1, 2 and 3 were immersed in a box containing 4373, 4611 and 5197 water molecules, respectively. At first, the energy of the water molecules was minimized, keeping the atoms of the SBs frozen. Then, a minimization of the whole system was performed by setting a convergence criterion on the gradient of 10^−4^ kcal mol^−1^ Å^−1^. Prior to starting the MD simulations, the system was equilibrated for 2 ns at 310 K in isocore conditions (NVT). Subsequently, 100 ns of MD simulations in an isothermal-isobaric ensemble were carried out at 310 K with a 2 fs time step (NPT). In the production runs, the systems were run with periodic boundary conditions. Van der Waals and short-range electrostatic interactions were estimated within a 20 Å cutoff. VMD 1.9.3 [33] was used for molecular visualization and for animating the trajectory data. The conformations adopted by the ligands were clustered by analyzing the MD trajectory frames and using the cpptraj module [34] of AMBER20 [29]. The MD frames were divided into clusters using the complete average linkage algorithm, and the geometries showing the lowest root mean square deviation (RMSD) to the cluster centers were acquired. The most representative geometries, populated for more than 10%, were then optimized, using the Gaussian16 program package [28]. Optimizations were conducted with water as the solvent using a self-consistent reaction field (SCRF) method, based on a polarizable continuum solvent model (PCM) [35] at the B3LYP/6-311+G(d) level [36,37]. Then, the possible complexes with metal ions Hg^2+^ and Pb^2+^ were constructed and optimized at the same level as above but using the effective core potential LanL2DZ for the central metal atom to correctly describe the geometries and the electronic properties of the chelating compounds [38]. Optimizations in the singlet (S = 0), triplet (S = 3) and quintet (S = 5) spin states were performed on the complexes. Vibrational frequencies were computed at the same level of theory to define the optimized structures as minima.

## 4. Conclusions

Schiff bases are very attractive ligands for metal coordination. They show the possibility of scaling up processes and an affinity for toxic HM ions (arsenic, cadmium, chromium, lead, mercury, etc.) with whom Schiff bases are able to form stable complexes. HMs are responsible of immeasurable biological damage, because they pollute the atmosphere, soil and water, as they are used in agriculture and industry. Mercury and lead ions, in particular, have effects on the gastrointestinal, respiratory and central nervous systems and lead to significant health problems in humans.

In this article, we reported a conformational analysis of three newly proposed SBs, **1**–**3**, as possible ligands for HMs through a combination of MD and DFT studies. The results highlight that all of the compounds have populated conformations that are suited to forming complexes with Hg^2+^ and Pb^2+^, which were optimized using the same DFT approach. The computational analysis confirmed that all of the ligands produce stable complexes with HMs, primarily using the *N*-imino moieties. Nevertheless, ligand **3**, showing a further ethylene diether chain with respect to **1** and **2**, presents the most promising coordination behavior, particularly with Pb^2+^. In fact, it provided a highly stable complex in which the two *N*-imino groups and the two oxygen atoms were on the same plane, forming the isosceles trapezoid base of a pyramid whose tip is lead. 

In conclusion, based on the modeling results, we will direct our future investigations towards the synthesis of **3** as a chemical sensor for lead ions. The data we obtained further underline the importance of modeling studies as tools that can be used to address targeted syntheses, avoiding the unnecessary consumption of reagents and solvents, which are generally harmful to the environment.

The results highlight the suitability of SBs as a scaffold for the detection of HMs in contaminated wastewater and offer insights for their potentially useful applications not only in environmental monitoring and remediation but also in the biomedical field.

## Figures and Tables

**Table 1:** Geometrical features, relative energies and equilibrium percentages of the selected conformations (populated for more than 10%) of SB **1**.

	ΔE [kcal mol^−1^]	%	d [Å] [a]	τ_1_ [°] [b]	τ_2_ [°] [c]	τ_3_ [°] [d]	τ_4_ [°] [e]	τ_5_ [°] [f]	τ_6_ [°] [g]
**1A**	0.00	27.5	6.81	−178	−119	−177	178	120	−177
**1B**	0.34	15.4	5.89	177	−119	−176	−66	127	177
**1C**	0.36	15.0	5.63	179	119	177	−73	−7	−177
**1D**	0.39	14.3	4.33	179	−127	67	67	−128	−176
**1E**	0.40	14.1	5.87	177	126	−66	−176	−119	177
**1F**	0.53	11.2	6.28	177	118	66	173	121	178
**others**	/	2.0	/	/	/	/	/	/	/

[a] d: -C=N…N=C-. [b] τ_1_: Car–N=C–CH_2_. [c] τ_2_: N=C–CH_2_-CH_2_. [d] τ_3_: =C–CH_2_-CH_2_-CH_2_. [e] τ_4_: CH_2_-CH_2_-CH_2_-C=. [f] τ_5_: CH_2_-CH_2_-C=N. [g] τ_6_: CH_2_-C=N-Car’.

**Table 2:** Geometrical features, relative energies and equilibrium percentages of the selected conformations of SB **2**.

	ΔE [kcal mol^−1^]	%	d [Å] [a]	τ_1_ [°] [b]	τ_2_ [°] [c]	τ_3_ [°] [d]	τ_4_ [°] [e]	τ_5_ [°] [f]	τ_6_ [°] [g]	τ_7_ [°] [h]
**2A**	0.00	45.4	6.24	−180	121	176	179	−177	−122	180
**2B**	0.23	30.5	5.44	180	−121	−176	179	67	−126	−179
**2C**	0.65	15.1	5.40	−180	121	177	178	64	123	180
**2D**	1.53	3.4	4.80	180	−122	179	65	61	123	180
**others**	/	6	/	/	/	/	/	/	/	/

[a] d: -C=N…N=C-. [b] τ_1_: =C–C=N–CH_2_. [c] τ_2_: C=N–CH_2_-CH_2_. [d] τ_3_: =N–CH_2_-CH_2_-CH_2_. [e] τ_4_: CH_2_–CH_2_-CH_2_-CH_2_. [f] τ_5_: CH_2_-CH_2_-CH_2_-N=. [g] τ_6_: CH_2_-CH_2_-N=C. [h] τ_7_: CH_2_-N=C-C=.

**Table 3:** Geometrical features, relative energies and equilibrium percentages of the selected conformations of SB **3**.

	ΔE [kcal mol^−1^]	%	τ_1_ [°] [a]	τ_2_ [°] [b]	τ_3_ [°] [c]	τ_4_ [°] [d]	τ_5_ [°] [e]	τ_6_ [°] [f]	τ_7_ [°] [g]	τ_8_ [°] [h]	τ_9_ [°] [i]	τ_10_ [°] [l]	τ_11_ [°] [m]
**3A**	0.00	66.4	180	−123	69	180	180	−70	179	179	175	124	180
**3B**	0.55	26.1	180	−124	−175	−179	180	−70	180	180	176	124	180
**3C**	1.35	6.9	180	−124	−175	−179	180	180	180	180	175	125	180
**others**	/	0.6	/	/	/	/	/	/	/	/	/	/	/

[a] τ_1_: =C–C=N–CH_2_. [b] τ_2_: C=N–CH_2_-CH_2_. [c] τ_3_: =N–CH_2_-CH_2_-O. [d] τ_4_: CH_2_–CH_2_-O-CH_2_. [e] τ_5_: CH_2_-O-CH_2_- CH_2_ [f] τ_6_: O-CH_2_- CH_2_-O. [g] τ_7_: CH_2_- CH_2_-O- CH_2_. [h] τ_8_: CH_2_-O- CH_2_-CH_2_. [i] τ_9_: [i] O-CH_2_- CH_2_-N=. [l] τ_10_: CH_2_- CH_2_-N=C. [m] τ_11_: CH_2_-N=C-C=.

**Table 4:** Relative energy values of complexes of SBs **1**–**3** with Hg^2+^ and Pb^2+^, respectively.

Name	ΔE (kcal/mol)
**CPX_1D_Hg^2+^**	0.00
**CPX_1D_Pb^2+^**	0.00
**CPX_2C_Hg^2+^**	2.38
**CPX_2D_Hg^2+^**	0.00
**CPX_2NEW_Pb^2+^**	0.00
**CPX_2D_Pb^2+^**	2.38
**CPX_3A_Hg^2+^**	7.90
**CPX_3B_Hg^2+^**	8.24
**CPX_3D_Hg^2+^**	0.00
**CPX_3A_Pb^2+^**	>20
**CPX_3B_Pb^2+^**	>20
**CPX_3D_Pb^2+^**	0.00

## Data Availability

Data is contained within the article.
